# Pathogenesis of *Mycobacterium bovis* Infection: the Badger Model As a Paradigm for Understanding Tuberculosis in Animals

**DOI:** 10.3389/fvets.2017.00247

**Published:** 2018-01-15

**Authors:** Eamonn Gormley, Leigh A. L. Corner

**Affiliations:** ^1^School of Veterinary Medicine, University College Dublin, Dublin, Ireland

**Keywords:** tuberculosis, *Mycobacterium bovis*, badgers, pathogenesis, infection, vaccination

## Abstract

Tuberculosis in animals is caused principally by infection with *Mycobacterium bovis* and the potential for transmission of infection to humans is often the fundamental driver for surveillance of disease in livestock and wild animals. However, with such a vast array of species susceptible to infection, it is often extremely difficult to gain a detailed understanding of the pathogenesis of infection––a key component of the epidemiology in all affected species. This is important because the development of disease control strategies in animals is determined chiefly by an understanding of the epidemiology of the disease. The most revealing data from which to formulate theories on pathogenesis are that observed in susceptible hosts infected by natural transmission. These data are gathered from detailed studies of the distribution of gross and histological lesions, and the presence and distribution of infection as determined by highly sensitive bacteriology procedures. The information can also be used to establish the baseline for evaluating experimental model systems. The European badger (*Meles meles*) is one of a very small number of wild animal hosts where detailed knowledge of the pathogenesis of *M. bovis* infection has been generated from observations in natural-infected animals. By drawing parallels from other animal species, an experimental badger infection model has also been established where infection of the lower respiratory tract mimics infection and the disease observed in natural-infected badgers. This has facilitated the development of diagnostic tests and testing of vaccines that have the potential to control the disease in badgers. In this review, we highlight the fundamental principles of how detailed knowledge of pathogenesis can be used to evaluate specific intervention strategies, and how the badger model may be a paradigm for understanding pathogenesis of tuberculosis in any affected wild animal species.

## Introduction

The presence of tuberculosis in wild animals has attracted scientific attention primarily because they are implicated in transmission of infection to livestock and other economically important species, and the risk of zoonotic transmission to humans. In Ireland and the UK, the European badger (*Meles meles*) is the principal wild animal species involved (1, 2). Elsewhere, wild boar (*Sus scrofa)* (3), goats (*Capra hircus*) (4) and species of deer, notably red deer (*Cervus elaphus*), fallow (*Dama dama*), and roe deer (*Capreolus capreolus*) are affected in continental Europe (5). In New Zealand, the brushtail possum (*Trichosurus vulpecula*) is the key species affected (6). In North America, white-tailed deer (*Odocoileus virginianus)* (7) elk (*Cervus canadensis*) (8) and bison (*Bison bison*) (9) are among the known reservoirs of infection. In Africa, many species of outstanding conservation merit are infected, posing a threat to the survival of local populations (10). With limited resources available to conduct surveillance programs, the gathering of basic information to develop an understanding of pathogenesis is rarely undertaken in natural-infected hosts. A key reason is often the physical size of the animal and the volume of tissues and specimens required to ensure maximum sensitivity and specificity of diagnostic procedures. Added problems include the availability of suitable samples and the potential for bias arising from misinterpretation of data because sampling is only from advanced disease cases or from cases identified by imperfect diagnostic tests.

There has been much written about the value of laboratory animals as surrogates in studying tuberculosis (11). This is mainly in the context of human tuberculosis where there is a drive to understand the host–pathogen interactions in great detail with a view to developing new therapies and vaccines (12). Those involved in trying to study the disease in a particular species are often reliant on information generated from laboratory animals, which may or may not be particularly relevant. Other than providing insights into pathogenesis at the animal level, laboratory animal models cannot contribute substantially toward understanding the epidemiology of human or animal tuberculosis at a population level. With the exception of the most commonly used laboratory animals, progress has also been hindered by the almost universal lack of reagents for specific animal species: this has constrained the development of diagnostic tests and limited the ability to understand how animals might respond to vaccines.

Nevertheless, animal models have been used extensively in tuberculosis research and proved invaluable in improving the understanding of pathogenesis and defining the subtle interactions between the pathogen and the host immune system (13, 14). The mouse model has been particularly useful and has revealed detailed functional information on many aspects of the host immunological responses to infection (15). The relative costs involved, the wide availability of immunological reagents, and the development of genetically modified lines have made the mouse model the pragmatic choice for many laboratories. However, the mouse is not considered to be a natural host for tuberculosis and study results often differ depending on the mouse strain used (16). This can be a cause for concern when extrapolating to different species. Other animal models including rabbits, guinea pigs, zebrafish, non-human primates, and cattle (a natural host of *M. bovis*) are all subject to the same constraints when applying the interpretation of the results across species (11). Notwithstanding the availability of reagents and the logistics and welfare of housing animals, there may be differences in the host response influenced by, for example, route of infection and pathogenesis. This all poses particular challenges for the study of tuberculosis in more exotic natural susceptible hosts and particular care needs to be taken to translate the results of studies from one model animal to another species. Key to this is deciding which pieces of information are relevant to the target species and how this can be used to develop a complete picture of the pathogenesis of infection. In Ireland, we have compared natural and experimental *M. bovis* infection models of badgers with a uniform level of postmortem examination, histology, and bacteriology. This has provided a unique opportunity to evaluate and gain insights into pathogenesis in both model systems.

## Tuberculosis in Badgers

The involvement of badgers in the epidemiology of tuberculosis in cattle is well established in Ireland and the UK (1, 17). Results from the four area badger removal trial in Ireland and the Randomized Badger Culling Trial (RBCT) in England provided evidence of a positive effect of badger culling on incidence rates of tuberculosis in associated cattle herds (18, 19). Arising from these studies, current policies to eradicate the disease are largely focused on surveillance testing of cattle supplemented with badger population control measures in areas of Ireland and England considered as high risk for cross-species transmission (1, 20). Analysis of *M. bovis* prevalence rates in approximately 5,000 badgers culled in Ireland in response to tuberculosis breakdowns in cattle herds has revealed a decrease in the overall prevalence from 26 to 11% between 2007 and 2011 (21). Nevertheless, large-scale culling is considered to be unsustainable in the long term, although it is recognized in Ireland, the UK, and other countries that eradication of tuberculosis in cattle is unlikely if the infection reservoir of *M. bovis* infection in badgers, and maybe other maintenance species, is not adequately addressed (22–24). The development of a vaccination strategy targeted at badgers is judged as a potentially feasible option; a key objective of vaccination is to reduce the transmission rate of infection within the badger population by reducing the level of susceptibility to infection or to alter the pathology of the infection in vaccinated badgers where protection is less than 100% to the extent that it decreases the rate of excretion of *M. bovis* and transmission to cattle (25). Until relatively recently there was limited detailed information relating to susceptibility of badgers to tuberculosis, and whether they were capable of resisting *M. bovis* infection through the generation of protective immune responses. A considerable body of research work has been carried out in Ireland and the UK to gain a greater understanding of the disease in badgers with the objective to develop and implement a vaccination strategy for badger populations (26). A critical step in this development stage is establishing a model system that closely mimics the natural infection state in free-living populations.

## Pathogenesis of Tuberculosis in Badgers

Insights into the pathogenesis and a baseline for the evaluation of experimental infection studies have been gained in badgers to a degree not previously undertaken in any other natural-infected species (27). Badgers are considered to be highly susceptible to *M. bovis* infection (28). As in many species, tuberculosis is principally a respiratory disease in badgers but in a natural-infected population there appears to be a second route of transmission by contamination of bite wounds, which is less prevalent but still significant. Pulmonary infection is established following inhalation of infectious aerosols and this leads to protracted progression toward a clinical state of disease (29). Aerosol infection results in a chronic disease and infected animals can express a variety of disease states ranging from latent subclinical infection (i.e., no visibly detectable lesions or clinical manifestations of disease) to moderate disease (with size-limited pulmonary and extrapulmonary lesions) and to severe overt disease with generalized pathology. In the majority of aerosol-infected badgers, however, infection remains latent and the proportion of badgers that develop generalized disease is small (29, 30). By contrast, bite wound infection results in a more rapid and progressive infection with typically generalized infection and lesions (29, 31). Despite the absence of lesions in the majority of infected animals, badgers with any state of infection may pose a risk of transmission to susceptible hosts where there is close and frequent contact.

**Figure 1:**
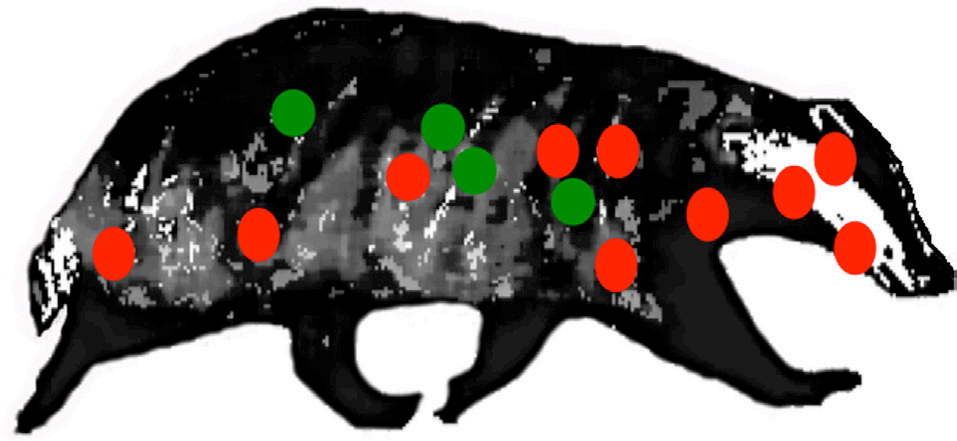
Location of lymph nodes (red) and visceral organs (green) examined for gross lesions, and samples for histological/bacteriological examination during systematic detailed postmortem of natural-infected wild badgers. Reproductive tract tissues (not shown) were also examined (31).

More refined insights into the pathology of disease have been revealed from detailed postmortem studies of culled badgers (31, 32). The sequence of events in the pathogenesis, including the early dissemination of infection from the lungs, is best demonstrated when the distribution of infection in the badgers is examined in a broad repertoire of anatomical sites (Figure 1). Gross visible lesions are commonly found in the thoracic cavity (lungs, tracheobronchial and mediastinal lymph nodes), with the head and body lymph nodes the most frequently affected extrathoracic sites. Visible lesions are scarce in the abdominal cavity; however, a broad range of tissues and organs may be infected. In badgers presenting with only a single site of infection, the distribution pattern is much the same as that in badgers with multiple infection sites. This could signify that host–pathogen interactions during the initial stages postinfection, rather than tissue predisposition, dissemination, or progression of disease, govern the infection distribution. Tuberculosis can also develop when bite wounds become infected with saliva containing infective bacilli. The spectrum of infected bite wounds can range from circumscribed subcutaneous granulomas, lacerated wounds draining abscesses, to large open ulcerated areas devoid of skin. The pathogenesis of infection following bite wound contamination differs from that after aerosol infection in that there is rapid progression of infection, a greater number of lesions, and wider distribution and severity of infection (27).

**Figure 2:**
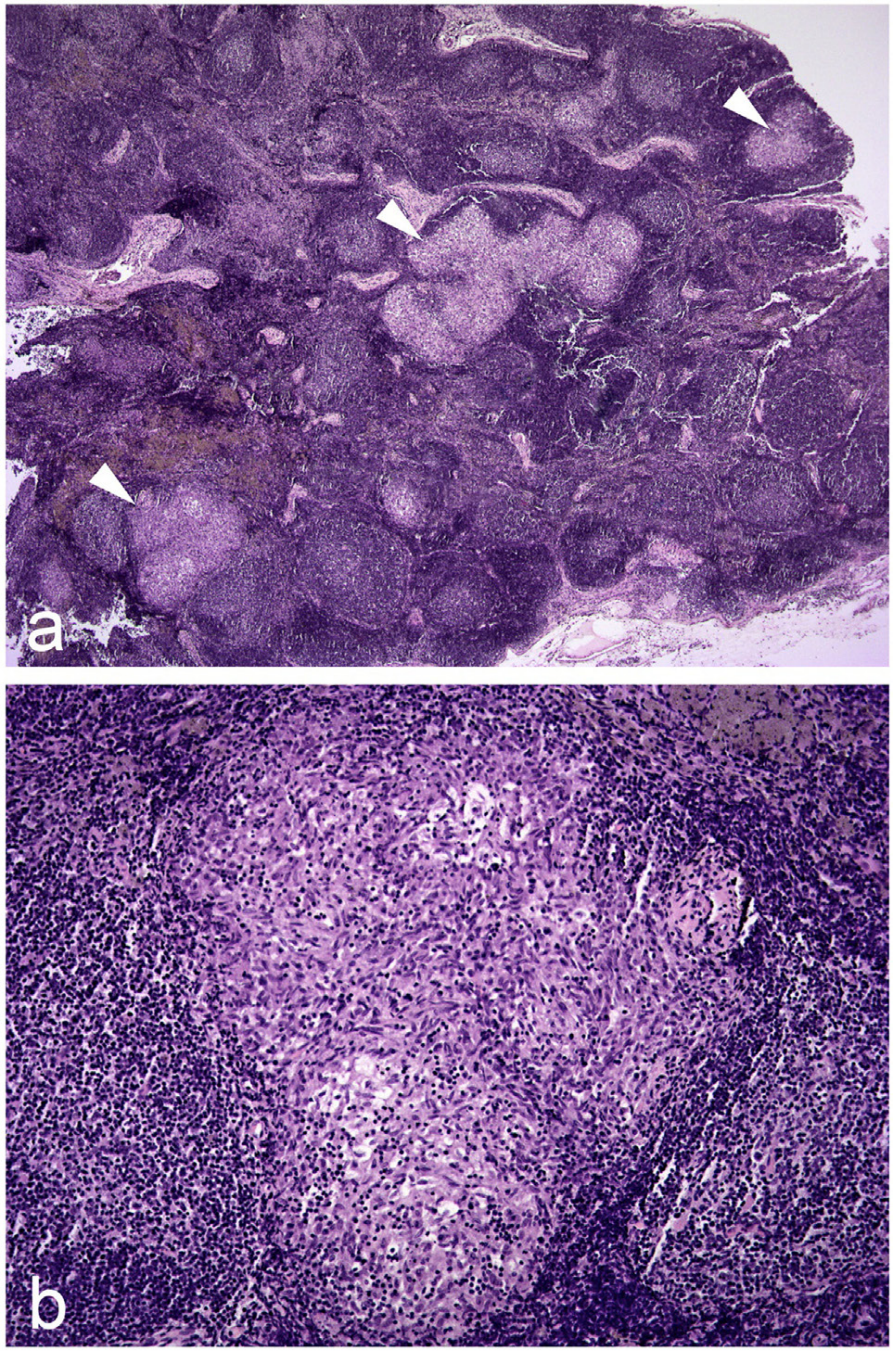
Photomicrographs illustrating **(A)** three granulomas of varying size indicated by white arrowheads in a hyperplastic/reactive lymph node of a badger; **(B)** higher magnification image of one of these granulomas. Hematoxylin and eosin stains, magnification ×20 **(A)** and ×100 **(B)**.

The presence of discrete tuberculous granulomas is the characteristic of infection in badgers as also occurs in other reservoir hosts including cattle (33), possums (34), and ferrets (35). These are composed largely of epithelioid cells, macrophages, and sporadic lymphocytes. Lesions are typically cellular and proliferative to a large extent, with limited necrosis, mineralization, or fibrosis (29, 36–38). Histologically, there is a wide variety in the size and structure of lesions present in an animal, and even in individual tissues (Figure 2). As the severity and size of lesions increase and the granulomas expands, the central mass of epithelioid cells increase and are enclosed by a peripheral rim of lymphocytes with the outer layer composed of macrophages and neutrophils, bounded by a narrow uneven fibroblast layer. As the infection progresses, lesions coalesce and may form large areas of necrosis and caseastion, sometimes associated with mineralization. The presence of acid-fast bacilli (AFB) becomes more common as the area of necrosis increases and then may be extracellular. In lung lesions, there may be erosion of bronchi and bronchioles walls, and AFB may be present in cellular debris in the lumina.

The specific immunological responses to infection with *M. bovis* can also offer important insights into key aspects of pathogenesis. However, measurement of these responses in badgers or other wild animals can be problematic not only because of a lack of reagents but also the requirement to repeatedly capture and collect samples from animals. In an experimental setting, this is feasible and it is possible to monitor changes in responses as the infection progresses (39, 40) As in all species studied, the predominant early specific immune response following *M. bovis* infection of badgers is T cell-mediated (CMI), leading to proliferation of T lymphocytes, secretion of interleukin-2 (IL-2) and release of pro-inflammatory cytokines including interferon-γ (IFN-γ) (41–43). The responses can be measured *ex vivo* following antigenic stimulation of blood or purified peripheral blood monocytes with tuberculin or the specific mycobacterial antigens ESAT-6/CFP10 cocktail. Where longitudinal studies have been conducted in wild badgers, the strength of the initial IFN-γ responses correlates with the progression of infection (44). Serological responses are associated with a later stage of infection when visible lesions are likely to be present and severity of disease is high (45). The immune-dominant serological antigen is restricted to the mycobacterial antigen MPB83, although the sensitivity of detection of antibodies recognizing this antigen is relatively low across the full spectrum of infection severity (46, 47). The innate immune response to infection with tuberculosis is regulated through the activity of macrophages; the key cells that are permissive for the growth of intracellular mycobacteria. Activated macrophages produce reactive nitrogen intermediates that are directly inhibitory for the growth of a wide range of intracellular organisms including *M. tuberculosis* (48) and *M. bovis* BCG (49). Nitric oxide (NO) is produced through oxidation of l-arginine (50) in a reaction catalyzed by an inducible nitric oxide synthase (iNOS) (51). However, studies in badgers have revealed that blood monocyte-derived macrophages do not produce NO or upregulate iNOS expression following *in vitro* activation of macrophages (52). This intriguing finding might imply that badgers should lack the ability to control infection *via* the innate response, though there is no strong pathological evidence to support this.

## Experimental Infection Models in Badgers

While the use of natural-infected animals has proved invaluable for investigating pathology across the broad spectrum of disease ranging from early infection to clinical disease, experimental models have the advantage of allowing study of the kinetics of disease progression and immunological responses starting from a fixed dose and a fixed point of time (28, 39, 40). This can facilitate reproduction of disease in a format necessary for the development of diagnostic tests and for evaluating vaccines. In order to understand fundamental aspects of pathogenesis, the experimental model needs to be framed around relevant infection routes, and plausible challenge doses that are reflective of natural transmission. The profile of infection that is generated should also be characteristic and consistent within the recognized spectrum of the naturally occurring disease. Principally, this requires evenness in the profile of lesion development and distribution of infection as found in natural *M. bovis* infection. In developing the badger infection model for tuberculosis in Ireland, the key factors that were considered to achieve this end were the choice of *M. bovis* strain, route of infection, the infective dose, and the kinetics of infection. The strain of *M. bovis* used for experimental infection was first isolated from a lesion in a clinically diseased badger (28). Spoligotyping revealed that the strain type was common in both infected badgers and cattle. In an initial study, the infective doses used were <10 colony forming units (CFU) (low dose), ~100 CFU (medium dose), and ~3,000 CFU (high dose) with delivery by the endobronchial route of infection, to mimic the dominant respiratory route of natural infection (28).

**Figure 3:**
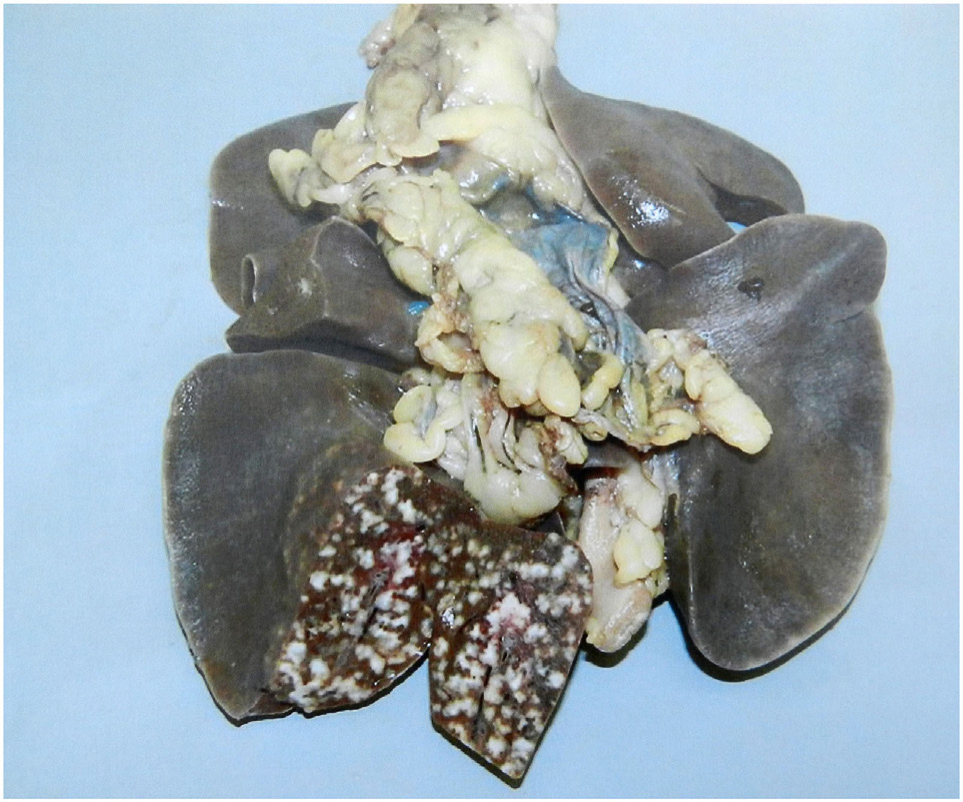
Experimental infection. Lesions of experimental pulmonary tuberculosis with miliary lesions of a uniform size in the inoculated lobe. In natural-infected badgers typically fewer pulmonary lesions are observed and they vary in size.

The results of this study showed that badgers were very susceptible to infection: all of the badgers had established infection across each of the doses used when animals were euthanized at 17 weeks postinfection (28). The results also demonstrated that the dose of *M. bovis* had a little effect on the distribution of infection but as the dose increased so did the rate of disease progression. There was a consistent profile of infection among the groups exposed to each dose according to the measures employed: distribution and number of lesions, severity score of gross lesions, distribution and number of infected tissues, levels of extrathoracic infection, and distribution and number of histological lesions. The inoculation resulted in a variety of infection states, ranging from latency (absence of gross lesions), to gross lesions in the lungs, draining and extrathoracic lymph nodes, and pleura. The experimental infections appeared to mimic the more severe end of the spectrum of lung disease found in natural-infected badgers in that pulmonary lesions ranged from 1- to 2-mm diameter discrete tubercles, to extensive miliary lesions, with consolidation and pervading caseation of lobes (Figure 3). Cavitation and liquefaction were not seen (37).

The CMI responses, as determined by antigen stimulation of peripheral blood mononuclear cells (PBMCs) with bovine purified protein derivative tuberculin (PPD-B), increased to levels associated with the infective dose and pathology recorded postmortem (39). The badgers infected with the highest dose of *M. bovis* developed the earliest immune responses at 3 weeks postinfection. In addition, the highest infective dose correlated with the most consistent CMI response. In those animals with latent infection the CMI responses were weak and indistinguishable from non-infected control animals over most of the study period. In contrast to the CMI response, the humoral antibody responses of the badgers were intermittent over the time period of the study and not strongly influenced by the dose of *M. bovis*.

The aim of the follow-up study was to describe pathological, bacteriological, and immunological changes over a 24-week infection period using only the high-dose rate (46, 53). Inoculation by the endobronchial route resulted in a non-progressive infection in most of the badgers, with limited dissemination of infection from the thoracic cavity. Where this occurred it was principally to the mesenteric and hepatic lymph nodes. However, there was a lack of uniformity in the progression of disease across time. There was contraction in the distribution of infection from that seen at 6 weeks to that at 12 weeks, but then progression of infection between 12 and 18 weeks but similar results for 18 and 24 weeks. The analysis suggested that changes in the distribution of infection between time points arose from non-uniform development of lesions within badgers and that local resolution of lesions may have also occurred. The CMI responses to PPD-B were first detectable 3 weeks after infection, and over the study period the responses of the PBMC to antigen stimulation with PPD-B and the specific antigen CFP-10 were positively associated with the presence of gross lesions in the infected badgers. There was no evidence of a direct correlation between the strength of the response and the severity or distribution of observed lesions. The serological response was largely restricted to MPB83 across all states of infection. An interesting finding from this study was the generation of immunoglobulin-G (IgG) recognition of MPB83 coincident with the CMI response at 3 weeks postchallenge.

A key feature of the badger experimental model has been the dose-dependent recreation of different states of infection, ranging from latency to clinical disease, all characteristic of natural infection. This is unique among natural hosts of infection in that each state in the badger can be defined by a combination of pathological and immunological parameters. In human tuberculosis, latency (skin test and IFN-γ immunological responses but no clinical or radiographic signs) is significant in the epidemiology of disease (54, 55). Latency is also an important feature in natural-infected badgers where, in a high proportion of infected badgers, infection is not associated with any clinical signs or gross pathology (31). The apparent lack of immunological responses in the natural or experimentally reproduced latency may reflect how the different immune systems control very low levels of infection, or strains of low virulence, e.g., bacillus of Calmette–Guérin (BCG), or may point to differences in pathogenesis of infection in the different hosts (56).

## BCG Vaccination and Pathogenesis of Disease in Badgers

Having established an experimental infection model that mimics the characteristics of moderate-to-severe natural disease, the studies progressed to evaluating BCG vaccine protection using the endobronchial experimental infection model (57, 58). A second objective was to determine if vaccination altered the pathogenesis of disease. The first study examined the effect of vaccination on the distribution of experimental infection. The BCG vaccine was administered in two different ways: subcutaneous injection (~5 × 10^5^ CFU) and application to mucosal membranes (nasal and conjunctival mucosa with total final vaccine dose of 4 × 10^5^ CFU). This latter route of immunization facilitated the delivery of BCG directly to an available mucosal surface, as a proxy route for delivery of an oral vaccine. With this protocol, the BCG was likely to be presented to the immune system *via* lymphoid tissues of the nasal cavity and/or conjunctiva. Inhaled aerosol particles may also have been exposed to the lymphoid tissues of the lower respiratory tract.

Following vaccination and endobronchial challenge (~10^4^ CFU *M. bovis*) infection was established in all vaccinates and in all of the control group. Compared with the controls, vaccinates had fewer sites of infection, sites of extrathoracic infection, and sites with gross lesions. In the group vaccinated by the subcutaneous routes, all these measures were lower than for those vaccinated by the mucosal route. Vaccination also altered the distribution of gross lesions following challenge with no extrathoracic gross lesions in the vaccinates. These effects were more pronounced for the subcutaneous group. It was 2 weeks after *M. bovis* challenge that the subcutaneous vaccinated group responded to PPD-B, whereas the remaining groups responded from 4 weeks postchallenge. Over the period of infection, the highest immune responses were recorded in the non-vaccinated/infected control group. The immune profiles observed also associated with lesion severity scores measured at postmortem in all groups. Vaccination was also expressed by a significant delay in seroconversion to MPB83, which correlated with the levels of severity of the disease in all groups. From an immunological perspective, an intriguing finding was the lack of CMI activity in response to vaccination, as measured by IFN-γ production. This has been found in other captive badger studies and there is evidence that it correlates positively with the dose of vaccine delivered (59). It is tempting to speculate that this lack of CMI activity following vaccination is mechanistically related to the absence of similar responses in latent-infected badgers. In both cases, it might suggest that innate T cells play a prominent role for limiting multiplication of bacilli and maintaining them at low numbers. In recent years, there is growing evidence of non-specific protective effects generated by vaccination with BCG and other live vaccines (60). It is thought to be mediated by cross-reactivity of T-cell responses to related and unrelated pathogens, and/or by “trained immunity” whereby the innate immune system is modified by epigenetic programming following initial exposure, in order to increase resistance to reinfection (61, 62). The repeated exposure of badgers to environmental mycobacteria may provide the conditions necessary for training of the innate immune system to maintain subsequent exposure to *M. bovis* as a latent infection in most cases, and to limit the requirement of a CMI response in response to BCG vaccination.

Further studies examined the effect of oral BCG vaccination (endo-esophageal instillation of ~10^8^ CFU BCG encapsulated in a lipid formulation) on protective effect and the distribution of infection after experimental challenge (63, 64). The predominant effect of BCG vaccination was seen as a reduction in the severity and number of gross lesions, decreased mycobacterial load in the lungs, and reduced number of sites of infection. However, vaccination did not alter the thoracic–extrathoracic pattern of infection. The CMI responses recorded in each of the vaccine groups were consistent with protection when expressed by pathology severity scores.

The most effective way of validating an experimental vaccination model is to compare it to vaccination of free-living animals in their natural environment. This allows vaccine protection to be assessed in a scenario of natural transmission. It also addresses an inherent limitation of captive animal vaccine studies where the infective dose and time of infection are controlled, leading to a relatively homogenous infection level in all animals from using relatively high-challenge doses. Two vaccine field trials have been carried out in badgers, in the UK and Ireland (65, 66). An injectable BCG vaccine for badgers was used in the UK trial and the results reported a 74% reduction in seropositivity among vaccinated badgers as compared with non-vaccinated badgers (65). Further analysis using a combination of diagnostic tests revealed a decreased risk of cubs testing positive as the proportion of adults vaccinated increased (67). In the Irish study, there was also a clear reduction in the rate of seroconversion among vaccinated badgers as compared with the non-vaccinated badgers, and in those vaccinated animals that did seroconvert, there was a significant time delay to when seroconversion occurred relative to the non-vaccinated badgers (66). The delayed time to seroconversion was consistent with that recorded in the captive badger vaccine studies where vaccine protection was also measured by time to seroconversion following endobronchial *M. bovis* challenge (33). A follow-up analysis using a different serology test confirmed that the proportion of seropositive animals was reduced in the vaccinated population compared with the non-vaccinated animals over the course of the trial (68).

## BCG Vaccination and Dissemination of Infection in Badgers

In all of the experimental vaccine studies, the protection levels generated by vaccination with BCG did not prevent establishment of infection; this may have reflected the severity of challenge resulting from endobronchial delivery of virulent *M. bovis*. However, vaccination did not appear to substantially change the pathogenesis of disease. One notable finding of the experimental vaccine protection studies was the level of dissemination of infection from the lung in all infected badgers including vaccinates. The principal measure of vaccine-induced protection was a reduction in the severity of thoracic and extrathoracic lesions rather than a reduction in the wider distribution of infection. The BCG vaccine is known to limit disseminated disease when delivered to children but is less effective in protecting adults against pulmonary disease (69–71). During the early innate stages of the immune response dissemination from the initial site of infection may occur before migration of the mycobacteria to a lymph node stimulates the development of a CMI response (72). Lesion development characteristic of tuberculosis will not commence until a sufficiently potent CMI response is generated, and this may take several weeks, dependent on the potency of the immune response and the severity of the infective dose. In the badger experimental model the average time to detect a measurable CMI response is 2–4 weeks (58, 63). During the period preceding this response, infected macrophages can use the lymphatic system to pass through the draining lymph nodes and circulate throughout the host. When a sufficiently dominant CMI response is induced, infected macrophages can become immobilized in lymph node tissue and further migration is restricted. Lesions can then develop and progress at sites where infected macrophages are resident, including those areas of the lung where infection was initiated. Following vaccination, antigen specific T-cells still take time to accumulate, allowing the infective bacilli to multiply and spread. In the badger model, the earliest time point for measuring postinfection responses is 2 weeks. The data indicate that responses in vaccinates appear around this time point and earlier than in non-vaccinates. Nevertheless, it still allows time for dissemination of infection to occur in the days following establishment of infection, by translocation of infected macrophages *via* the lymphatic system or the blood stream. Local dissemination may occur by movement of infected macrophages within tissues or following accumulation of infected debris in the lymphatics, or blood vessels, airways, renal or gastrointestinal system (29). This may partly explain why vaccination is particularly successful in reducing the severity of disease but has limited impact on distribution of infection.

## Experimental Infection Models for Domestic Species and Wild Animal Tuberculosis

Studies in natural susceptible hosts and experimental studies in captive natural susceptible hosts are indispensable in advancing the understanding of the pathogenesis of tuberculosis. This in turn can generate confidence in the interpretation of results from experimental vaccine—challenge studies. In many domestic species, e.g., cattle, goats, and deer, the presentation of tuberculosis is similar to that observed in the greater majority of infected humans in that it is a chronic, slowly progressive disease with pathology predominantly confined to the lower respiratory tract, with associated cellular immune responses (73, 74). However, the occurrence and prevalence of latency in these species has not been clearly established and is difficult to identify in either natural-infected or experimentally infected animals.

Many of the pathogenesis studies and infection models developed in livestock and wild animals have been motivated by the desire to develop vaccines to control inter-species spread of tuberculosis (75). With this in mind, development of a reliable experimental infection model is important for a number of reasons including (a) the need to establish infection with an appropriate dose, (b) keeping the dose biologically plausible, (c) obtaining a balance ensuring infection is successful in all animals including vaccinates, (d) ensuring that the infection route used and the pathology generated is at least within the spectrum of the disease in its natural state, and (e) there are appropriate and measurable parameters for quantifying protective immunity.

Unlike laboratory animal model systems, livestock and wild animals are usually natural hosts for infection and are relatively outbred. Therefore, replicating the pathogenesis of natural infection might be confounded by the differing environmental conditions experienced by free-living and captive animals, and also different levels of natural susceptibility to infection. Experimental infection of domestic cattle with low doses of *M. bovis* (10^2^ to 10^3^ CFU) by intratracheal/endobronchial inoculation or by aerosol-generating systems has resulted in lesions similar to those detected in the lungs and associated lymph nodes of natural-infected animals (76, 77). A natural challenge system of housing tuberculosis-free cattle with natural-infected animals has been presented as a biologically plausible challenge system but with a highly variable degree of success (78, 79). Experimental infections using endobronchial (*M. caprae*) or aerosol delivery (*M. bovis*) have been carried out in goats (80, 81). In both cases, the distribution of lesions mimicked those seen in natural infection. Further studies in goats experimentally infected with selected members of the *M. tuberculosis* complex (*M. bovis, M. caprae, M. tuberculosis*) revealed different clinical outcomes with lesion scores ranking highest with *M. bovis*, then *M. caprae* and *M. tuberculosis* (51). These results highlight a good example of host tropism associated with closely related mycobacterial species. Sheep have also been experimentally infected with *M. caprae* by the endotracheal route resulting in granulomatous caseous and necrotizing lesions in the lung and associated lymph nodes, typically found in natural cases of sheep tuberculosis (82). In addition, there were similar measured immunological, pathological, and bacteriological parameters as found in experimental *M. caprae* infection of goats. In deer naturally infected with *M. bovis*, the distribution of lesions differs from other domestic animals in that lesions are found predominantly in the retropharyngeal lymph nodes followed by lung and thoracic lymph nodes. Experimental infection models in deer routinely target inoculation of the tonsillar crypts to mimic natural infection and lesion distribution (83, 84). Additional studies in white-tailed deer have demonstrated that dissemination from the tonsil is an infrequent event involving low numbers of bacilli (85).

Ferrets (*Mustela Furo*) are susceptible to infection with *M. bovis* and are considered as part of the epidemiology of *M. bovis* transmission in New Zealand (86). As mustelids related to badgers, they offer some advantages as a model animal species in that they are available from licensed suppliers, and are relatively easy to house and maintain in captivity. Experimental infection models have been established where *M. bovis* was delivered to captive ferrets by the oral or aerosol route (35, 87). In both models, infection was found in the thoracic cavity and also in the mesenteric lymph nodes. The mechanism of dissemination from the primary site of infection in both models is not clearly understood. To date, experimental challenge by the endobronchial route has not been reported; this would allow for direct comparison of different aspects of pathogenesis with badgers, which might reveal subtle differences in specific host–pathogen interactions influencing dissemination.

The wild boar (*Sus scrofa*) is considered as a key maintenance host for tuberculosis in Spain with prevalence rates >50% in areas with high-density populations (88). Vaccination is being explored as a potential strategy to control the level of disease in these wild animals. In the first challenge study, a field isolate of *M. bovis* was delivered to boar by the oropharyngeal route using a range of infective doses between 10^2^ and 10^6^ CFU (89). All four challenged wild-boar-developed lesions and severe generalized lesions were observed in two animals exposed to the highest dose: this was not considered typical of the lesion distribution encountered in natural infections.

Routes of infection not observed in natural-infected wild animals have been used in experimental infections to achieve particular outcomes. Such alternative inoculation routes have been used to experimentally infect New Zealand brushtail possums to counter the high susceptibility of these animals to experimental infection and the rapid progression of disease. Delivery of *M. bovis via* conjunctival instillation resulted in established infection in a dose-dependent manner (90). The infection progressed slowly in the possums, generating palpable lesions in superficial lymph node lesions, and widespread distribution of macroscopic and microscopic lesions, all characteristics of the disease in wild, natural-infected possums. Percutaneous inoculation of *M. bovis* suspension into the paws resulted in gross lesions in superficial lymph nodes as is observed in natural-infected possums (91). As with the conjunctival challenge route, percutaneous infection prolonged the postchallenge survival period. However, although these two alternative routes of infection do result in gross lesion distribution as seen in natural-infected wild possums, they significantly underestimate the distribution of infection as seen when more detailed postmortem examination procedures are employed (6). Natural transmission between infected and susceptible captive possums was also investigated as a possible method of evaluating vaccination in captive possums but proved unsuitable (92). The rate of *M. bovis* transmission was lowest when animals were mixed at random; however, transmission increased significantly with mixing of more sociable possums. This indicated that transmission was influenced by the proximity of susceptible and infected possums leading to increased frequency and duration of social interactions.

Though the presence of visible lesions, whether in experimentally infected or in natural-infected animals, can point toward the likely route of natural infection, caution must be taken in interpreting such data when only a limited number of infection parameters are measured. This highlights one of the difficulties when working with relatively large animal species to understand pathogenesis. The sensitivity of detection of infection can be severely compromised in large animals, such as cattle and deer, simply because of the difficulty of detecting small lesions or infection in large organs and lymph nodes (93). This can lead to biases when analysis is based on visible lesion detection. In all animals, there is the possibility of microscopic lesions or small bacteriological loads existing in the carcase but too small to detect, this being an increasing probability as the mass of the animal increases. Sampling from natural-infected populations can also compound this when only animals considered likely to have large lesions, or strong reactors to the skin test are recruited for analysis. The problem here is that it represents a relatively late stage of disease progression and the distribution of lesions may not reflect the early stage of pathogenesis and the likely route of infection.

Even when using natural-infected animals across a broad spectrum of disease, the interpretation can be subjected to bias if insensitive protocols for bacteriology and histology are employed. In studying the disease in natural-infected badgers, we tried to minimize these biases by randomly sampling from the infected population and by using sensitive bacteriological and histological procedures on a predetermined set of 36 tissues covering a wide range of anatomically diverse tissue samples in each animal. In addition, the tissues for bacteriology were collected aseptically so that the bacteriological detection of infection was maximized and tissues were individually cultured for the same reason (31). This intensity of pathological investigation has not been repeated in any other natural-infected animal species but for the badger, at least, it provides a reference point to compare natural-infected animals with those from experimental infection studies.

## Concluding Remarks

The pathological and bacteriological examination of natural-infected badgers with tuberculosis when using sensitive postmortem examination procedures has provided an important and essential baseline for understanding the pathogenesis and epidemiology of tuberculosis in this species. It has also provided the framework for developing experimental infection studies, which has allowed the evaluation of diagnostic assays and the measurement of vaccine efficacy. There is a growing awareness that *M. bovis* infection is naturally endemic in a diverse range of domestic and wild animals, as well as in humans. Experimentally, at least, it seems that most animal species are susceptible to infection. With the continuous threat of infection spreading from wild animals to livestock and the risk of onward transmission to humans, the continued improvement in knowledge of pathogenesis and epidemiology will serve to lessen the transmission risks into the future.

## Author Contributions

EG and LC contributed equally to this review including critical analysis of published data and preparation of the manuscript.

## Conflict of Interest Statement

The authors declare that the research was conducted in the absence of any commercial or financial relationships that could be construed as a potential conflict of interest.

## References

[1] Ní Bhuachalla DN Corner LAL More SJ Gormley E The role of badgers in the epidemiology of *Mycobacterium bovis* infection (tuberculosis) in cattle in the United Kingdom and the Republic of Ireland: current perspectives on control strategies. Vet Med Res Rep (2015) 6:27–38. 10.2147/Vmrr.S53643 PMC6067767 30101094

[2] Delahay RJ Langton S Smith GC Clifton-Hadley RS Cheeseman CL The spatio-temporal distribution of *Mycobacterium bovis* (bovine tuberculosis) infection in a high-density badger population. J Anim Ecol (2000) 69(3):428–41. 10.1046/j.1365-2656.2000.00406.x

[3] Vicente J Hofle U Garrido JM Fernandez-De-Mera IG Juste R Barral M Wild boar and red deer display high prevalences of tuberculosis-like lesions in Spain. Vet Res (2006) 37(1):107–19. 10.1051/vetres:2005044 16336928

[4] Rodriguez S Bezos J Romero B de Juan L Alvarez J Castellanos E *Mycobacterium caprae* infection in livestock and wildlife, Spain. Emerg Infect Dis (2011) 17(3):532–5. 10.3201/eid1703.100618 21392452 PMC3165998

[5] Gortazar C Delahay RJ Mcdonald RA Boadella M Wilson GJ Gavier-Widen D The status of tuberculosis in European wild mammals. Mammal Rev (2012) 42(3):193–206. 10.1111/j.1365-2907.2011.00191.x

[6] Jackson R Cooke MM Coleman JD Morris RS. Naturally occurring tuberculosis caused by *Mycobacterium bovis* in brushtail possums (*Trichosurus vulpecula*): I. An epidemiological analysis of lesion distribution. N Z Vet J (1995) 43(7):306–14. 10.1080/00480169./1995.35911 16031871

[7] Waters WR Palmer MV Whipple DL Slaughter RE Jones SL. Immune responses of white-tailed deer (*Odocoileus virginianus*) to *Mycobacterium bovis* BCG vaccination. J Wildl Dis (2004) 40(1):66–78. 10.7589/0090-3558-40.1.66 15137490

[8] Thoen CO Quinn WJ Miller LD Stackhouse LL Newcomb BF Ferrell JM. Mycobacterium bovis infection in North American elk (*Cervus elaphus)*. J Vet Diagn Invest (1992) 4(4):423–7. 10.1177/104063879200400410 1457545

[9] Himsworth CG Elkin BT Nishi JS Neimanis AS Wobeser GA Turcotte C An outbreak of bovine tuberculosis in an intensively managed conservation herd of wild bison in the Northwest Territories. Can Vet J (2010) 51(6):593–7. 20808568 PMC2871352

[10] Renwick AR White PC Bengis RG. Bovine tuberculosis in southern African wildlife: a multi-species host-pathogen system. Epidemiol Infect (2007) 135(4):529–40. 10.1017/S0950268806007205 16959052 PMC2870607

[11] Williams A Orme IM Animal models of tuberculosis: an overview. Microbiol Spectr (2016) 4(4):1–11. 10.1128/microbiolspec.TBTB2-0004-2015 27726810

[12] Fonseca KL Rodrigues PNS Olsson IAS Saraiva M. Experimental study of tuberculosis: from animal models to complex cell systems and organoids. PLoS Pathog (2017) 13(8):e1006421. 10.1371/journal.ppat.1006421 28817682 PMC5560521

[13] Cooper AM Dalton DK Stewart TA Griffin JP Russell DG Orme IM. Disseminated tuberculosis in interferon gamma gene-disrupted mice. J Exp Med (1993) 178(6):2243–7. 10.1084/jem.178.6.2243 8245795 PMC2191280

[14] Flynn JL Chan J Triebold KJ Dalton DK Stewart TA Bloom BR. An essential role for interferon gamma in resistance to *Mycobacterium tuberculosis* infection. J Exp Med (1993) 178(6):2249–54. 10.1084/jem.178.6.2249 7504064 PMC2191274

[15] Orme IM. The mouse as a useful model of tuberculosis. Tuberculosis (2003) 83(1–3):112–5. 10.1016/S1472-9792(02)00069-0 12758199

[16] Smith CM Proulx MK Olive AJ Laddy D Mishra BB Moss C Tuberculosis susceptibility and vaccine protection are independently controlled by host genotype. MBio (2016) 7(5):e01516. 10.1128/mBio.01516-16 27651361 PMC5030360

[17] Clifton-Hadley RS Wilesmith JW Richards MS Upton P Johnston S. The occurrence of *Mycobacterium bovis* infection in cattle in and around an area subject to extensive badger (*Meles meles*) control. Epidemiol Infect (1995) 114(1):179–93. 10.1017/S0950268800052031 7867737 PMC2271337

[18] Griffin JM Williams DH Kelly GE Clegg TA O’Boyle I Collins JD The impact of badger removal on the control of tuberculosis in cattle herds in Ireland. Prev Vet Med (2005) 67(4):237–66. 10.1016/j.prevetmed.2004.10.009 15748755

[19] Donnelly CA Wei G Johnston WT Cox DR Woodroffe R Bourne FJ Impacts of widespread badger culling on cattle tuberculosis: concluding analyses from a large-scale field trial. Int J Infect Dis (2007) 11(4):300–8. 10.1016/j.ijid.2007.04.001 17566777

[20] Brunton LA Donnelly CA O’Connor H Prosser A Ashfield S Ashton A Assessing the effects of the first 2 years of industry-led badger culling in England on the incidence of bovine tuberculosis in cattle in 2013-2015. Ecol Evol (2017) 7(18):7213–30. 10.1002/ece3.3254 28944012 PMC5606900

[21] Byrne AW Kenny K Fogarty U O’Keeffe JJ More SJ McGrath G Spatial and temporal analyses of metrics of tuberculosis infection in badgers (*Meles meles)* from the Republic of Ireland: trends in apparent prevalence. Prev Vet Med (2015) 122(3):345–54. 10.1016/j.prevetmed.2015.10.013 26556049

[22] Gormley E Corner LA. Control strategies for wildlife tuberculosis in Ireland. Transbound Emerg Dis (2013) 60(Suppl 1):128–35. 10.1111/tbed.12095 24171858

[23] Chambers MA Carter SP Wilson GJ Jones G Brown E Hewinson RG Vaccination against tuberculosis in badgers and cattle: an overview of the challenges, developments and current research priorities in Great Britain. Vet Rec (2014) 175(4):90–6. 10.1136/vr.102581 25059963

[24] Che’Amat A Armenteros JA Gonzalez-Barrio D Lima JF Diez-Delgado I Barasona JA Is targeted removal a suitable means for tuberculosis control in wild boar? Prev Vet Med (2016) 135:132–5. 10.1016/j.prevetmed.2016.11.002 27843020

[25] Gormley E Collins JD. The development of wildlife control strategies for eradication of tuberculosis in cattle in Ireland. Tuber Lung Dis (2000) 80(4/5):229–36. 10.1054/tuld.2000.0250 11052912

[26] Chambers MA Gormley E Corner LAL Smith GC Delahay RJ Tuberculosis in badgers (*Meles meles*). In: Mukundan H Chambers MA Waters WR Larsen MH, editors. Tuberculosis, Leprosy and Mycobacterial Diseases of Man and Animals: The Many Hosts of Mycobacteria. CABI (2015). p. 296–312. 10.1079/9781780643960.0296

[27] Corner LA Murphy D Gormley E *Mycobacterium bovis* infection in the Eurasian badger (*Meles meles*): the disease, pathogenesis, epidemiology and control. J Comparative Path (2011) 144(1):1–24. 10.1016/j.jcpa.2010.10.003 21131004

[28] Corner LA Costello E Lesellier S O’Meara D Sleeman DP Gormley E. Experimental tuberculosis in the European badger (*Meles meles*) after endobronchial inoculation of *Mycobacterium bovis*: I. Pathology and bacteriology. Res Vet Sci (2007) 83(1):53–62. 10.1016/j.rvsc.2006.10.016 17197004

[29] Gallagher J Clifton-Hadley RS. Tuberculosis in badgers; a review of the disease and its significance for other animals. Res Vet Sci (2000) 69(3):203–17. 10.1053/rvsc.2000.0422 11124091

[30] Gallagher J Monies R Gavier-Widen M Rule B. Role of infected, non-diseased badgers in the pathogenesis of tuberculosis in the badger. Vet Rec (1998) 142(26):710–4. 10.1136/vr.142.26.710 9682428

[31] Corner LA O’Meara D Costello E Lesellier S Gormley E. The distribution of *Mycobacterium bovis* infection in naturally infected badgers. Vet J (2012) 194(2):166–72. 10.1016/j.tvjl.2012.03.013 22542391

[32] Murphy D Gormley E Costello E O’Meara D Corner LA. The prevalence and distribution of *Mycobacterium bovis* infection in European badgers (*Meles meles*) as determined by enhanced post mortem examination and bacteriological culture. Res Vet Sci (2010) 88(1):1–5. 10.1016/j.rvsc.2009.05.020 19545882

[33] Liebana E Johnson L Gough J Durr P Jahans K Clifton-Hadley R Pathology of naturally occurring bovine tuberculosis in England and Wales. Vet J (2008) 176(3):354–60. 10.1016/j.tvjl.2007.07.001 17728162

[34] Cooke MM Jackson R Coleman JD Alley MR. Naturally occurring tuberculosis caused by *Mycobacterium bovis* in brushtail possums (*Trichosurus vulpecula*): II. Pathology. N Z Vet J (1995) 43(7):315–21. 10.1080/00480169./1995.35912 16031872

[35] Cross ML Labes RE Mackintosh CG Oral infection of ferrets with virulent *Mycobacterium bovis* or *Mycobacterium avium*: susceptibility, pathogenesis and immune response. J Comp Path (2000) 123(1):15–21. 10.1053/jcpa.1999.0379 10906251

[36] Gavier-Widen D Chambers MA Palmer N Newell DG Hewinson RG. Pathology of natural *Mycobacterium bovis* infection in European badgers (*Meles meles*) and its relationship with bacterial excretion. Vet Rec (2001) 148(10):299–304. 10.1136/vr.148.10.299 11315135

[37] Nolan A Wilesmith JW Tuberculosis in badgers (*Meles meles*). Vet Microbiol (1994) 40(1–2):179–91. 10.1016/0378-1135(94)90054-X 8073624

[38] Gallagher J Muirhead RH Burn KJ. Tuberculosis in wild badgers (*Meles meles*) in Gloucestershire: pathology. Vet Rec (1976) 98:9–14. 10.1136/vr.98.1.9 769293

[39] Lesellier S Corner L Costello E Sleeman P Lyashchenko KP Greenwald R Immunological responses following experimental endobronchial infection of badgers (*Meles meles*) with different doses of *Mycobacterium bovis*. Vet Immunol Immunopathol (2009) 127(1–2):174–80. 10.1016/j.vetimm.2008.09.012 18986710

[40] Chambers MA Aldwell F Williams GA Palmer S Gowtage S Ashford R The effect of oral vaccination with *Mycobacterium bov*is BCG on the development of tuberculosis in captive European badgers (*Meles meles*). Front Cell Infect Microbiol (2017) 7:6 10.3389/fcimb.2017.00006 28174695 PMC5258709

[41] Dalley D Chambers MA Cockle P Pressling W Gavier-Widen D Hewinson RG A lymphocyte transformation assay for the detection of *Mycobacterium bovis* infection in the Eurasian badger (*Meles meles*). Vet Immunol Immunopathol (1999) 70(1–2):85–94. 10.1016/S0165-2427(99)00072-0 10507289

[42] Southey A Costello E Gormley E. Detection of *Mycobacterium bovis* infection and production of interleukin-2 by *in vitro* stimulation of badger lymphocytes. Vet Immunol Immunopathol (2002) 87(1–2):73–8. 10.1016/S0165-2427(02)00129-0 12052344

[43] Dalley D Dave D Lesellier S Palmer S Crawshaw TR Hewinson RG Development and evaluation of a gamma-interferon assay for tuberculosis in badgers (*Meles meles*). Tuberculosis (2008) 88:235–43. 10.1016/j.tube.2007.11.001 18083067

[44] Tomlinson AJ Chambers MA McDonald RA Delahay RJ Association of quantitative interferon-gamma responses with the progression of naturally acquired *Mycobacterium bovis* infection in wild European badgers (*Meles meles*). Immunol (2015) 144(2):263–70. 10.1111/imm.12369 PMC4298420 25109384

[45] Chambers MA Crawshaw T Waterhouse S Delahay R Hewinson RG Lyashchenko KP. Validation of the BrockTB stat-pak assay for detection of tuberculosis in Eurasian badgers (*Meles meles*) and influence of disease severity on diagnostic accuracy. J Clin Micro (2008) 46(4):1498–500. 10.1128/JCM.02117-07 18272706 PMC2292970

[46] Lesellier S Corner L Costello E Sleeman P Lyashchenko K Greenwald R Antigen specific immunological responses of badgers (*Meles meles*) experimentally infected with *Mycobacterium bovis*. Vet Immunol Immunopathol (2008) 122(1–2):35–45. 10.1016/j.vetimm.2007.11.005 18082897

[47] Lyashchenko KP Greenwald R Esfandiari J Chambers MA Vicente J Gortazar C Animal-side serologic assay for rapid detection of *Mycobacterium bovis* infection in multiple species of free-ranging wildlife. Vet Microbiol (2008) 132(3–4):283–92. 10.1016/j.vetmic.2008.05.029 18602770

[48] Denis M. Interferon-gamma-treated murine macrophages inhibit growth of tubercle bacilli via the generation of reactive nitrogen intermediates. Cell Immunol (1991) 132(1):150–7. 10.1016/0008-8749(91)90014-3 1905984

[49] Flesch IE Kaufmann SH. Mechanisms involved in mycobacterial growth inhibition by gamma interferon-activated bone marrow macrophages: role of reactive nitrogen intermediates. Infect Immun (1991) 59(9):3213–8. 1908829 10.1128/iai.59.9.3213-3218.1991 PMC258155

[50] Granger DL Hibbs JB Jr Perfect JR Durack DT. Metabolic fate of l-arginine in relation to microbiostatic capability of murine macrophages. J Clin Invest (1990) 85(1):264–73. 10.1172/JCI114422 2404026 PMC296414

[51] Bekker LG Freeman S Murray PJ Ryffel B Kaplan G. TNF-alpha controls intracellular mycobacterial growth by both inducible nitric oxide synthase-dependent and inducible nitric oxide synthase-independent pathways. J Immunol (2001) 166(11):6728–34. 10.4049/jimmunol.166.1.6728 11359829

[52] Bilham K Boyd AC Preston SG Buesching CD Newman C Macdonald DW Badger macrophages fail to produce nitric oxide, a key anti-mycobacterial effector molecule. Sci Rep (2017) 7:45470. 10.1038/srep45470 28382943 PMC5382539

[53] Corner LA Costello E Lesellier S O’Meara D Gormley E. Experimental tuberculosis in the European badger (*Meles meles*) after endobronchial inoculation with *Mycobacterium bovis*: II. Progression of infection. Res Vet Sci (2008) 85(3):481–90. 10.1016/j.rvsc.2008.03.003 18433810

[54] Esmail H Barry CE III Young DB Wilkinson RJ. The ongoing challenge of latent tuberculosis. Philos Trans R Soc Lond B Biol Sci (2014) 369(1645):20130437. 10.1098/rstb.2013.0437 24821923 PMC4024230

[55] Getahun H Matteelli A Chaisson RE Raviglione M Latent *Mycobacterium tuberculosis* infection. N Engl J Med (2015) 372(22):2127–35. 10.1056/NEJMra1405427 26017823

[56] Cardona PJ. A dynamic reinfection hypothesis of latent tuberculosis infection. Infection (2009) 37(2):80–6. 10.1007/s15010-008-8087-y 19308318

[57] Corner LA Costello E Lesellier S O’Meara D Gormley E. Vaccination of European badgers (*Meles meles*) with BCG by the subcutaneous and mucosal routes induces protective immunity against endobronchial challenge with *Mycobacterium bovis*. Tuberculosis (2008) 88:601–9. 10.1016/j.tube.2008.03.002 18468490

[58] Lesellier S Corner L Costello E Lyashchenko K Greenwald R Esfandiari J Immunological responses and protective immunity in BCG vaccinated badgers following endobronchial infection with *Mycobacterium bovis*. Vaccine (2009) 27(3):402–9. 10.1016/j.vaccine.2008.10.068 19010372

[59] Lesellier S Palmer S Dalley DJ Dave D Johnson L Hewinson RG The safety and immunogenicity of Bacillus Calmette-Guerin (BCG) vaccine in European badgers (*Meles meles*). Vet Immunol Immunopathol (2006) 112(1–2):24–37. 10.1016/j.vetimm.2006.03.009 16687176

[60] Kleinnijenhuis J Quintin J Preijers F Joosten LA Ifrim DC Saeed S Bacille Calmette-Guerin induces NOD2-dependent nonspecific protection from reinfection via epigenetic reprogramming of monocytes. Proc Natl Acad Sci U S A (2012) 109(43):17537–42. 10.1073/pnas.1202870109 22988082 PMC3491454

[61] Benn CS Netea MG Selin LK Aaby P A small jab – a big effect: nonspecific immunomodulation by vaccines. Trends Immunol (2013) 34(9):431–9. 10.1016/j.it.2013.04.004 23680130

[62] Bekkering S Blok BA Joosten LA Riksen NP van Crevel R Netea MG. In vitro experimental model of trained innate immunity in human primary monocytes. Clin Vacc Immunol (2016) 23(12):926–33. 10.1128/CVI.00349-16 27733422 PMC5139603

[63] Corner LA Costello E O’Meara D Lesellier S Aldwell FE Singh M Oral vaccination of badgers (*Meles meles*) with BCG and protective immunity against endobronchial challenge with *Mycobacterium bovis*. Vaccine (2010) 28(38):6265–72. 10.1016/j.vaccine.2010.06.120 20637774

[64] Murphy D Costello E Aldwell FE Lesellier S Chambers MA Fitzsimons T Oral vaccination of badgers (*Meles meles*) against tuberculosis: comparison of the protection generated by BCG vaccine strains Pasteur and Danish. Vet J (2014) 200(3):362–7. 10.1016/j.tvjl.2014.02.031 24792450

[65] Chambers MA Rogers F Delahay RJ Lesellier S Ashford R Dalley D Bacillus Calmette-Guerin vaccination reduces the severity and progression of tuberculosis in badgers. Proc Biol Sci (2011) 278(1713):1913–20. 10.1098/rspb.2010.1953 21123260 PMC3097825

[66] Gormley E Ni Bhuachalla D O’Keeffe J Murphy D Aldwell FE Fitzsimons T Oral vaccination of free-living badgers (*Meles meles*) with Bacille Calmette Guerin (BCG) vaccine confers protection against tuberculosis. PLoS One (2017) 12(1):e0168851 10.1371/journal.pone.0168851 28121981 PMC5266210

[67] Carter SP Chambers MA Rushton SP Shirley MD Schuchert P Pietravalle S BCG vaccination reduces risk of tuberculosis infection in vaccinated badgers and unvaccinated badger cubs. PLoS One (2012) 7(12):e49833. 10.1371/journal.pone.0049833 23251352 PMC3521029

[68] Aznar I Frankena K More SJ O’Keeffe J McGrath G de Jong MCM Quantification of *Mycobacterium bovis* transmission in a badger vaccine field trial. Prev Vet Med (2018) 149:29–37. 10.1016/j.prevetmed.2017.10.010 29290298

[69] Trunz BB Fine P Dye C. Effect of BCG vaccination on childhood tuberculous meningitis and miliary tuberculosis worldwide: a meta-analysis and assessment of cost-effectiveness. Lancet (2006) 367(9517):1173–80. 10.1016/S0140-6736(06)68507-3 16616560

[70] Walker V Selby G Wacogne I Does neonatal BCG vaccination protect against tuberculous meningitis? Arch Dis Child (2006) 91(9):789–91. 10.1136/adc.2006.098459 16923863 PMC2082927

[71] Colditz GA Brewer TF Berkey CS Wilson ME Burdick E Fineberg HV Efficacy of BCG vaccine in the prevention of tuberculosis. Meta-analysis of the published literature. JAMA (1994) 271(9):698–702. 10.1001/jama.1994.03510330076038 8309034

[72] Chackerian AA Alt JM Perera TV Dascher CC Behar SM. Dissemination of *Mycobacterium tuberculosis* is influenced by host factors and precedes the initiation of T-cell immunity. Infect Immun (2002) 70(8):4501–9. 10.1128/IAI.70.8.4501-4509.2002 12117962 PMC128141

[73] Waters WR Palmer MV. *Mycobacterium bovis* infection of cattle and white-tailed deer: translational research of relevance to human tuberculosis. ILAR J (2015) 56(1):26–43. 10.1093/ilar/ilv001 25991696

[74] Buddle BM Vordermeier HM Hewinson RG. Experimental infection models of tuberculosis in domestic livestock. Microbiol Spectr (2016) 4(4):1–15. 10.1128/microbiolspec.TBTB2-0017-2016 27726786

[75] Buddle BM Parlane NA Wedlock DN Heiser A. Overview of vaccination trials for control of tuberculosis in cattle, wildlife and humans. Transbound Emerg Dis (2013) 60(Suppl 1):136–46. 10.1111/tbed.12092 24171859

[76] Buddle BM de Lisle GW Pfeffer A Aldwell FE. Immunological responses and protection against *Mycobacterium bovis* in calves vaccinated with a low dose of BCG. Vaccine (1995) 13(12):1123–30. 10.1016/0264-410X(94)00055-R 7491820

[77] Palmer MV Waters WR Whipple DL. Aerosol delivery of virulent *Mycobacterium bovis* to cattle. Tuberculosis (2002) 82(6):275–82. 10.1054/tube.2002.0341 12623270

[78] Khatri BL Coad M Clifford DJ Hewinson RG Whelan AO Vordermeier HM A natural-transmission model of bovine tuberculosis provides novel disease insights. Vet Rec (2012) 171(18). 10.1136/vr.101072 22935562

[79] Ameni G Vordermeier M Aseffa A Young DB Hewinson RG. Field evaluation of the efficacy of *Mycobacterium bovis* bacillus Calmette-Guerin against bovine tuberculosis in neonatal calves in Ethiopia. Clin Vacc Immunol (2010) 17(10):1533–8. 10.1128/CVI.00222-10 20719984 PMC2953002

[80] de Val Perez B Lopez-Soria S Nofrarias M Martin M Vordermeier HM Villarreal-Ramos B Experimental model of tuberculosis in the domestic goat after endobronchial infection with *Mycobacterium caprae*. Clin Vacc Immunol (2011) 18(11):1872–81. 10.1128/CVI.05323-11 21880849 PMC3209027

[81] Gonzalez-Juarrero M Bosco-Lauth A Podell B Soffler C Brooks E Izzo A Experimental aerosol *Mycobacterium bovis* model of infection in goats. Tuberculosis (2013) 93(5):558–64. 10.1016/j.tube.2013.05.006 23850102

[82] Balseiro A Altuzarra R Vidal E Moll X Espada Y Sevilla IA Assessment of BCG and inactivated *Mycobacterium bovis* vaccines in an experimental tuberculosis infection model in sheep. PLoS One (2017) 12(7):e0180546. 10.1371/journal.pone.0180546 28678885 PMC5498051

[83] Mackintosh CG Qureshi T Waldrup K Labes RE Dodds KG Griffin JF. Genetic resistance to experimental infection with *Mycobacterium bovis* in red deer (*Cervus elaphus*). Infect Immun (2000) 68(3):1620–5. 10.1128/IAI.68.3.1620-1625.2000 10678981 PMC97322

[84] Palmer MV Waters WR Whipple DL. Lesion development in white-tailed deer (*Odocoileus virginianus*) experimentally infected with *Mycobacterium bovis*. Vet Pathol (2002) 39(3):334–40. 10.1354/vp.39-3-334 12014497

[85] Thacker TC Palmer MV Robbe-Austerman S Stuber TP Waters WR. Anatomical distribution of *Mycobacterium bovis* genotypes in experimentally infected white-tailed deer. Vet Microbiol (2015) 180(1–2):75–81. 10.1016/j.vetmic.2015.07.006 26243696

[86] Ragg JR Waldrup KA Moller H. The distribution of gross lesions of tuberculosis caused by *Mycobacterium bovis* in feral ferrets (*Mustela furo*) from Otago, New Zealand. N Z Vet J (1995) 43(7):338–41. 10.1080/00480169./1995.35916 16031876

[87] McCallan L Corbett D Andersen PL Aagaard C McMurray D Barry C A new experimental infection model in ferrets based on aerosolised *Mycobacterium bovis*. Vet Med Int (2011) 2011:981410. 10.4061/2011/981410 21547237 PMC3087619

[88] Vicente J Barasona JA Acevedo P Ruiz-Fons JF Boadella M Diez-Delgado I Temporal trend of tuberculosis in wild ungulates from Mediterranean Spain. Transbound Emerg Dis (2013) 60(Suppl 1):92–103. 10.1111/tbed.12167 24171854

[89] Ballesteros C Garrido JM Vicente J Romero B Galindo RC Minguijon E First data on Eurasian wild boar response to oral immunization with BCG and challenge with a *Mycobacterium bovis* field strain. Vaccine (2009) 27(48):6662–8. 10.1016/j.vaccine.2009.08.095 19747578

[90] Corner LA Buddle BM Morris RS. Experimental infection of brushtail possums (*Trichosurus vulpecula*) with *Mycobacterium bovis* by conjunctival instillation. Vet J (2003) 166(2):177–84. 10.1016/S1090-0233(02)00311-8 12902183

[91] Nugent G Whitford EJ Yockney I Perry M Tompkins DM Holtslag N Percutaneous interdigital injection of *Mycobacterium bovis* as a model for tuberculous lesion development in wild brushtail possums (*Trichosurus vulpecula*). J Comp Path (2013) 148(1):33–42. 10.1016/j.jcpa.2012.05.006 22749650

[92] Corner LA Pfeiffer DU de Lisle GW Morris RS Buddle BM. Natural transmission of *Mycobacterium bovis* infection in captive brushtail possums (*Trichosurus vulpecula*). N Z Vet J (2002) 50(4):154–62. 10.1080/00480169.2002.36302 16032262

[93] Corner LA. Post mortem diagnosis of *Mycobacterium bovis* infection in cattle. Vet Microbiol (1994) 40(1–2):53–63. 10.1016/0378-1135(94)90046-9 8073629

